# Atypical Cutaneous Manifestations in Adult Onset Still's Disease

**DOI:** 10.1155/2016/4835147

**Published:** 2016-02-15

**Authors:** Champa Nataraja, Hedley Griffiths

**Affiliations:** ^1^Barwon Health, Bellerine Street, Geelong, VIC 3220, Australia; ^2^Barwon Rheumatology Service, 156 Bellerine Street, Geelong, VIC 3220, Australia

## Abstract

Adult Onset Still's Disease (AOSD), an adult variant of systemic onset juvenile idiopathic arthritis, is a rare systemic inflammatory disorder of unknown aetiology. The rarity of this disease is associated with low index of suspicion and delayed diagnosis in patients suffering from it and in the presence of atypical features the diagnosis can be further challenging. This is a case report on a 24-year-old woman, who was a diagnostic dilemma for 2 years due to the nonspecific symptoms of recurrent fever, generalized maculopapular persistent pruritic and tender rash, and polyarthralgia. She was initially diagnosed as leukocytoclastic vasculitis on a skin biopsy and was managed by a dermatologist with various medications including NSAIDs, hydroxychloroquine, dapsone, colchicine, cyclosporine, and high doses of oral steroids with minimal response. Subsequently, she has had multiple admissions with similar symptoms with raised inflammatory markers and negative septic workup. On one occasion, her iron study revealed hyperferritinaemia which led to the suspicion of AOSD. Once the rheumatic fever and infectious, malignant, autoimmune, and lymphoproliferative disorders were excluded, she was diagnosed as probable AOSD and managed successfully with IL-1 (interleukin-1) receptor antagonist, Anakinra, with remarkable and lasting response both clinically and biochemically.

## 1. Introduction

AOSD is a systemic, inflammatory disorder characterized by spiking fevers, arthritis/arthralgia, and an evanescent salmon-pink maculopapular rash, frequently accompanied by sore throat, myalgia, lymphadenopathies, splenomegaly, and neutrophilic leucocytosis. The aetiology of this disease is unknown with equal distribution between the sexes. There is a bimodal age distribution, with one peak between the ages of 15 and 25 and the second peak between the ages of 35 and 45. The clinical course can be divided into monophasic, intermittent, and chronic pattern. Systemic manifestations predominate in the two former forms while articular involvement predominates in patients with chronic form. AOSD has been associated with markedly elevated serum ferritin level (>3000 ng/mL) in as much as 70% of patients. As there is no diagnostic test, it is a diagnosis of exclusion. The most popular Yamaguchi et al.'s criteria [1] have been proposed for diagnosis which involves four major and five minor criteria. The diagnosis requires the presence of five features with at least two being major criteria. Treatment usually involves NSAIDs, corticosteroids, steroid sparing agents including methotrexate, biological agents including IL-1 receptor antagonist, Anakinra, Canakinumab, IL-6 antagonist, Tocilizumab, and TNF-alpha inhibitors. It is a life-long treatment and prognosis is variable, depending on treatment response.

## 2. Case Report

**Figure 1:**
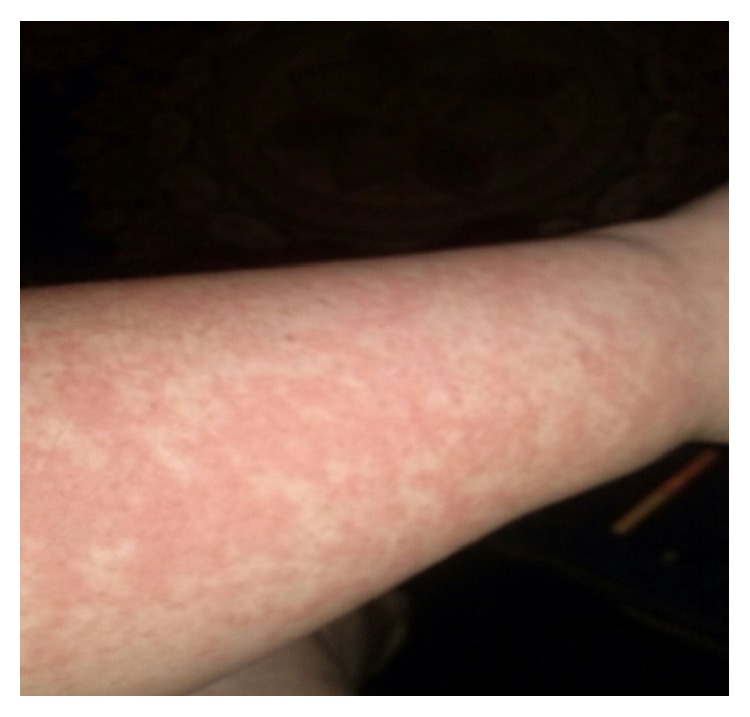
Maculopapular rash involving left forearm.

**Figure 2:**
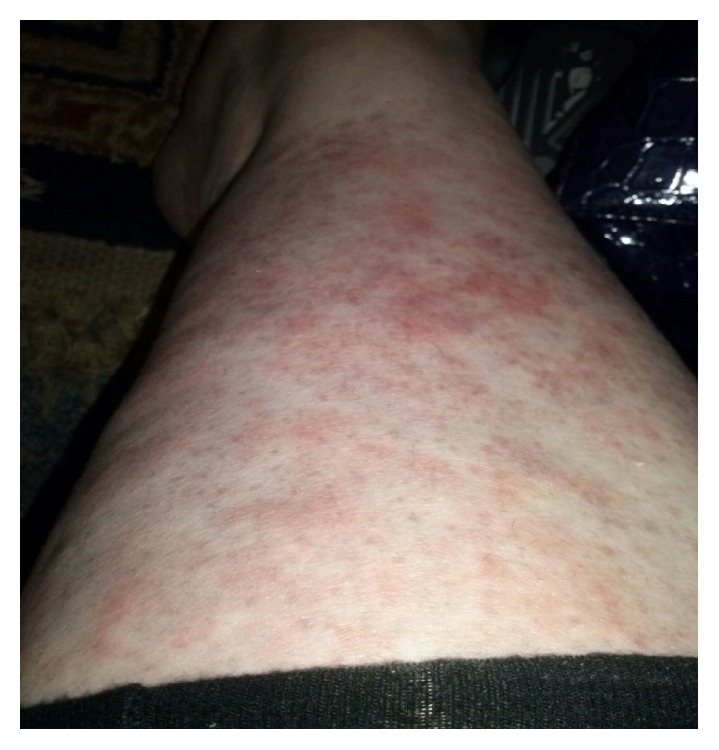
Maculopapular rash involving right leg.

**Figure 3:**
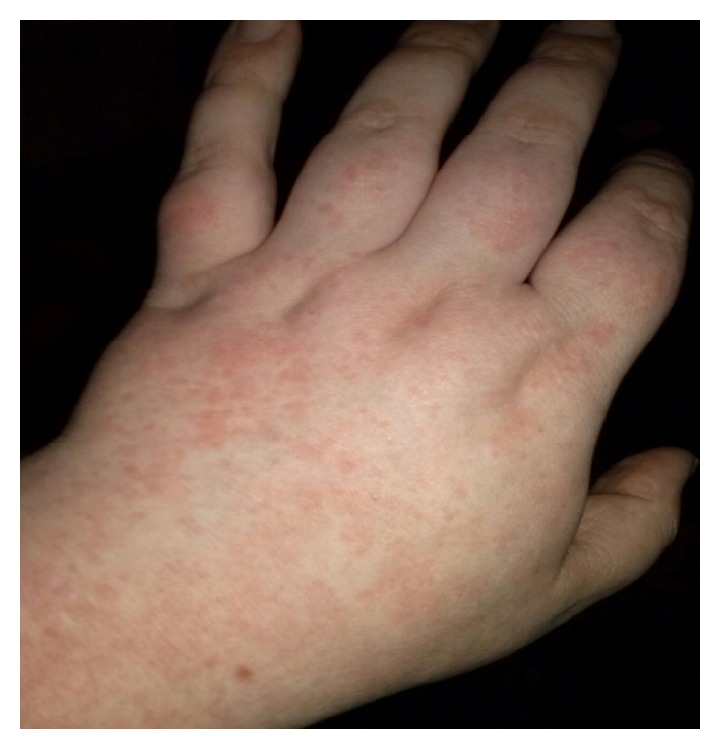
Maculopapular rash involving left hand.

A 24-year-old woman initially presented 2 years ago with the widespread pruritic, burning maculopapular rash predominantly involving limbs (Figures 1, 2, and 3), torso, chest, and back associated with high grade fever and polyarthralgia. The rash was tender to palpate. It was diagnosed as leukocytoclastic vasculitis on a skin biopsy and was managed by dermatologist with various medications including antihistamines, NSAIDs, high dose of oral steroids and topical steroids, dapsone, hydroxychloroquine, colchicine, and cyclosporine with minimal response. Her symptoms were intermittent with symptom-free period between the episodes. She was seen once by the rheumatologists in those 2 years, and unfortunately during that consultation, she was symptom-free. She chose to follow up with the dermatologists from then on. She has had several admissions to hospital with similar symptoms and the repeat skin biopsy only revealed features of drug reaction without any evidence of vasculitis. Her last admission was 12 months ago with severe symptoms of widespread persistent, pruritic, and painful rash with high temperatures >39, raised inflammatory markers, and widespread arthralgia; however, there was no evidence of synovitis clinically.

Her laboratory findings were as shown in Table 1.

Her serological test results were as follows: antinuclear antibody (ANA) titre, negative; anti-ds-DNA, 9 units (normal value < 30); ANCA, negative; complements (C3, C4), normal; extractable nuclear antigen (ENA), negative; lupus inhibitor, not detected; immunoglobulin levels were normal with normal cryoglobulin levels.

She had negative hepatitis and HIV serological tests. Thyroid function test was found to be normal. Her extensive septic workup including chest X-ray and urine and multiple blood culture sensitivities were negative; cardiac echocardiogram was normal. PET scan revealed extensive lymphadenopathy involving lymph nodes above and below the diaphragms associated with splenomegaly and hepatomegaly. She subsequently underwent cervical excisional lymph node biopsy which revealed mixed nonspecific paracortical and follicular hyperplasia and excluded lymphoma. There was no family history to suggest a genetic origin to her symptoms. Based on the clinical, laboratory, and pathological examinations, the infectious, malignant, lymphoproliferative, and autoimmune disorders were excluded and probable diagnosis of AOSD was made as per Yamaguchi et al.'s criteria [1]. In our patient, she has had presence of three major criteria (fever > 39, arthralgia lasting 2 weeks or more, and leucocytosis) with an atypical rash and four minor criteria (sore throat, lymphadenopathy, splenomegaly, hepatomegaly, and negative tests for ANA and rheumatoid factor).

Our patient was treated with IL-1 antagonist, Anakinra 100 mg s/c daily (due to the poor response to high dose steroids and other DMARDs in the past). Notable clinical and laboratory regression was observed during her check a month later [2]. Her arthralgia and rash regressed. Laboratory results were serum ferritin 66 micrograms/L, ESR 3 mm/hr, CRP 7.5 mg/L, and haemoglobin 137 g/L. Outpatient follow-up of the patient continues.

## 3. Discussion

AOSD is rare systemic inflammatory disorder of unknown aetiology. In 1971, the term Adult Still's Disease was used to describe a series of adult patients who had features similar to the children with systemic juvenile idiopathic arthritis and did not fulfil criteria for classic rheumatoid arthritis. The diagnosis is often clinical and often diagnosis of exclusion. The classic skin rash in AOSD is an evanescent, salmon-coloured, macular, or maculopapular cutaneous eruption that is usually nonpruritic and tends to occur with fever and disappear during afebrile episode. The classical features on skin biopsy demonstrate perivascular inflammation of the superficial dermis with invasion of lymphocytes and histiocytes and immunohistochemistry sometimes positive for complement and immunoglobulin [3].

Leukocytoclastic vasculitis is a variant of cutaneous vasculitis. The characteristic features include maculopapular eruption, often painful or burning sensation with pruritus. Some authors state that leukocytoclastic vasculitis is vasculitis limited to skin, and some state that systemic involvement can be mild to moderate [4]. Many medications like penicillins, cephalosporins, sulphonamides, phenytoin, and allopurinol and infections like viral and bacterial infections can cause cutaneous vasculitis. In our case, there were no new medications commenced prior to the appearance of symptoms and all the serological tests for viral and bacterial infections were negative including autoimmune screen. The typical histological features in leukocytoclastic vasculitis include disruption of small vessels by inflammatory cells, deposition of fibrin within the lumen, and/or vessel wall coupled with nuclear debris that allows for the confident recognition of small vessel, mostly neutrophilic vasculitis [5]. The first biopsy in our patient showed features consistent with leukocytoclastic vasculitis as described and the second skin biopsy showed mild spongiotic reaction pattern with dermal inflammation including eosinophils, features suggestive of a drug reaction.

In our case, the rash was persistent, pruritic and tender, and recalcitrant to most medications, which is not a classical feature of AOSD. Even though the initial skin biopsy showed vasculitic picture, subsequent biopsies revealed urticarial reaction/drug reaction without any evidence of vasculitis. The nature of the rash and positive skin biopsy for vasculitis initially delayed the diagnosis of AOSD in our case. Coexistence of leukocytoclastic vasculitis and AOSD is very rare [6, 7]; however, persistent pruritic rash with AOSD is not uncommon [8, 9].

Recent advances revealed a pivotal role of proinflammatory cytokines such as tumour necrosis factor-alpha, IL-1, IL-6, and IL-18 in disease pathogenesis, giving rise to the development of novel targeted therapies aiming at optimal disease control [10].

## 4. Conclusion

In the initial stages of the disease, the diagnosis of AOSD is challenging due to the absence of specific or diagnostic clinical, serological, and pathological findings. The skin lesions of AOSD are important for the correct diagnosis. Persistent pruritic, erythematous maculopapules and plaques may be an atypical initial presentation of AOSD; therefore, it is important to consider various cutaneous manifestations of AOSD for an early and correct diagnosis [7].

## Figures and Tables

**Table 1:** 

Laboratory tests	Patient's value	Normal value
Full blood count		
WCC (×10^9^)	16.6	4.0–11.0
Neutrophils (×10^9^)	12.6	2.0–8.0
Band cells	2.7	0.0–0.5
Hemoglobin (g/L)	104	115–170
Platelets (×10^9^)	239	150–500
MCV (fl)	64	80–98
MCHC (pg)	20.4	26–34
Liver function tests		
ALP (u/L)	125	8–252
GGT (u/L)	88	<30
ALT (u/L)	12	<45
Albumin (g/L)	26	34–50
Iron studies		
Serum Iron (micromol/L)	5	7–30
Ferritin (microgm/L)	6198	8–252
Acute phase reactants		
CRP (mg/L)	350	<2.9
ESR (mm/hr)	51	0–12
LDH (u/L)	794	80–230

## Abbreviations

AOSD: Adult Onset Still's Disease
NSAIDs: Nonsteroidal anti-inflammatory drugs
IL-1: Interleukin-1
IL-6: Interleukin-6
DMARDs: Disease modifying antirheumatoid drugs.
